# Development and validation of a comprehensive prevention-focused intervention package for problematic digital technology use among youth: a multi-site study protocol

**DOI:** 10.3389/fpsyt.2026.1804588

**Published:** 2026-05-08

**Authors:** Yatan Pal Singh Balhara, Vishal Dhiman, Siddharth Sarkar, Rajeev Ranjan, Rachna Bhargava, Ragul Ganesh, Shivanand Kattimani, Bichitra Nanda Patra, Syed Hubbe Ali, Audrey Gogoi

**Affiliations:** 1 Behavioral Addictions Clinic (BAC) and Centre for Advanced Research on Addictive Behaviours (CARAB), National Drug Dependence Treatment Centre (NDDTC), All India Institute of Medical Sciences (AIIMS), New Delhi, India; 2 AIIMS, Rishikesh, India; 3 AIIMS, New Delhi, India; 4 AIIMS, Patna, India; 5 Jawaharlal Institute of Postgraduate Medical Education & Research (JIPMER), Puducherry, India; 6 Department of Psychiatry, AIIMS, New Delhi, India; 7 United Nations International Children's Emergency Fund (UNICEF), New Delhi, India; 8 National Drug Dependence Treatment Centre (NDDTC), AIIMS, New Delhi, India

**Keywords:** digital well-being, internet and smartphone addiction, prevention-focused intervention, problematic digital technology use, youth mental health

## Abstract

**Background:**

Problematic use of digital technology among children, adolescents, and young adults is associated with adverse health, behavioural, interpersonal, social, academic and vocational outcomes. Most existing research focuses on treatment oriented interventions. Prevention focused interventions are limited. This is especially true for the low- and middle-income countries. There is a need for structured prevention approaches that involve youth, parents, and teachers.

**Objectives:**

This study aims to develop and validate a comprehensive package of prevention-focused interventions targeted at problematic use of digital technology among youth.

**Methods:**

The study will be conducted across six sites in India. It will use a sequential mixed-methods design. Literature review, stakeholder interviews, and expert consensus shall be used for intervention development. This will be guided by established frameworks for complex interventions. Validation will be carried out using a quasi-experimental pre–post design. Quantitative measures will assess changes in knowledge, skills, confidence, and decision-making, as well as feasibility and acceptability. Qualitative methods will be used to assess engagement, delivery quality, and contextual factors.

**Expected outcomes:**

The study will lead to a modular prevention-focused intervention package with evidence of feasibility and acceptability. Findings will inform future larger scale implementation and evaluations.

**Conclusion:**

This protocol outlines a structured approach to development of a prevention-focused intervention targeted at problematic digital technology use among youth. The focus on prevention, stakeholder involvement, and real-world settings supports relevance for public health practice and policy.

**Clinical trial registration:**

https://ctri.nic.in/Clinicaltrials/login.php, identifier CTRI2026/03/105278.

## 1 Introduction

Digital technology is embedded in the daily lives of children, adolescents, and young adults. Digital devices and the internet related platforms support learning, work, leisure, and social contact. However, their poorly regulated use is linked to various adverse outcomes. These risks increase with early exposure and high daily screen time.

Problematic use of digital technology is a growing public health concern among the youth (article under review) (1–3). It has been associated with higher levels of depression, anxiety, stress, sleep disturbance, and reduced quality of life (4–9). Problematic use of digital technology is associated with higher levels of depression, anxiety, stress, sleep disturbance, and reduced quality of life (4–6). Patterns characterised by impaired control and continued use despite harm resemble behavioural addictions and may co-occur with substance use and suicidal risk (10, 11). Excessive engagement is also associated with reduced attention, academic decline, social withdrawal, and decreased offline interaction (12). Neuroimaging studies report altered brain connectivity related to self-regulation and reward processing among the youth. This increases their vulnerability to such problems (13).

Problematic use of digital technology refers to using technology in a pattern that leads to impaired control, increasing priority given to the behaviour, and continued engagement despite negative consequences. It may be associated with significant distress, dysfunction or both. This can manifest in the form of use of digital devices (online and offline) and the internet. It can occur in various contexts including use of the internet, smartphones, gaming, social media, gambling, shopping/buying, Over-The-Top (OTT) content watching, pornography watching, and excessive screen time. These are all potentially addictive behaviours. While many of these have not been listed as specified diagnostic categories, International Classification of Diseases (ICD-11) makes provision for listing these under the residual categories in the section on addictive behaviours (14).

Current responses to problematic digital technology use focus mainly on treatment after problems emerge (15). Preventive efforts are limited. Many programs address single behaviours or platforms and often exclude parents and teachers. Few interventions are designed for use across age groups or adapted to varied social and educational settings. Evidence from low- and middle-income countries (LMIC) remains limited (16).

In India, rapid growth in digital access has outpaced guidance on healthy use. Educational institutions and families often lack structured tools to support balanced digital technology use related behaviours. Differences in access, supervision, and digital skills add to this gap. Preventive interventions that are practical, theory-based, scalable, and suited to real-world educational and family settings are needed (14). However, such interventions remain limited in India (17, 18). International experience suggests that prevention of problematic digital technology use is most effective when education is combined with behaviour-change techniques and parental engagement. A recent rapid systematic review emphasized the importance of combining educational, behavioural, and environmental strategies (19). Systematic reviews of school-based prevention programmes recommend multi-component, theory-driven approaches. It is recommended that these programmes should incorporate skills training, self-regulation strategies, and stakeholder involvement (20). Programmes such as Be Internet Awesome and Stop and Play programme have been evaluated and found to be effective (21, 22).

This study protocol describes a structured process to develop and validate a comprehensive package of preventive interventions for problematic digital technology use among youth. The package targets youth, parents, and teachers through role-specific components. Development is guided by established frameworks for complex interventions and informed by literature review, stakeholder input, and expert consensus.

This study has been proposed as part of the Centre for Advanced Research on Addictive Behaviours (CAR-AB) (14). CAR-AB was established at All India Institute of Medical Sciences (AIIMS), New Delhi, in collaboration with leaders from health, public health, technology and education sectors and with the funding support from the Indian Council of Medical Research (ICMR). CAR-AB aims to establish a scientific, systematic, and sustainable framework for addressing addictive behaviours, and promoting digital and overall well-being among Indian youth. CAR-AB envisions promotion of safe digital technology use and enhanced digital well-being among Indian youth.

The current study will use a phased design. It will include intervention development, feasibility testing, and validation using mixed methods approach. This approach is intended to lead to systematic refinement and early assessment of feasibility, acceptability, and perceived impact.

### 1.1 Objectives

The primary objective of the study is to develop and validate a comprehensive, prevention-focused package of interventions targeting problematic digital technology use among youth. The intervention also aims to address the associated stress, depression, anxiety and addiction. The target population includes the children, adolescents, and young adults. The intervention package will include components for youth, parents and teachers. The study also aims to assess the feasibility, acceptability, and perceived usefulness of the intervention package across diverse settings.

## 2 Methods

### 2.1 Study setting

Six sites will be included in the study. They represent different regions of India. These include New Delhi, Bhopal (Madhya Pradesh), Patna (Bihar), Puducherry, Rishikesh (Uttarakhand), and Shillong (Meghalaya).

### 2.2 Study design

The study will be conducted in sequential phases. The first phase will focus on intervention development. This phase shall use an exploratory qualitative design. It shall also include a structured literature review. The second phase will involve validity testing of the developed intervention package using a quasi-experimental mixed-methods pre–post design. The participants will serve as their own controls. This design will allow assessment of feasibility, acceptability, and preliminary perceived impact, of the intervention (23, 24).

### 2.3 Eligibility criteria

#### 2.3.1 Inclusion criteria

##### 2.3.1.1 For intervention development

The participants will include school and college students aged 12 years or older, parents, and teachers. In addition, mental health professionals, addiction experts, child and adolescent mental health experts, public health experts, and program or policy experts shall be included. Those providing informed consent or assent, as appropriate, shall be included in the study. Participants who decline consent will be excluded.

##### 2.3.1.2 For validity testing

The participants will include school and college students aged 12 years or older, parents, and teachers. Those providing informed consent or assent, as appropriate, shall be included in the study. Participants who decline consent/assent will be excluded.

### 2.4 Sample size

For intervention development: for focus group discussions (FGDs) and in-depth interviews (IDIs) recruitment will continue until data saturation is achieved for each stakeholder group. We anticipate conducting at least one FGD per stakeholder group per site, resulting in a minimum of 18 FGDs across six sites. Additional FGDs may be conducted if data saturation is not achieved. We anticipate conducting approximately 5–8 IDIs per stakeholder category across sites. Recruitment will continue until thematic saturation is achieved.

For feasibility testing: A minimum of five participants per group (i.e. youth, parents, and teachers) will be recruited from one school and one college at each site, resulting in a total sample of at least 30 participants. These participants will be different from the ones which were included in the sample for the development of the intervention package.

For validity testing: The minimum required sample size was calculated to detect a medium effect size of 0.5 with a two-sided alpha of 0.05 and a power of 95%. This resulted in a requirement of 53 participants per group. Accounting for an anticipated dropout rate of approximately 10%, at least 59 participants per group will be recruited. With three participant groups, the total planned sample size will be a minimum of 354.

### 2.5 Recruitment

For intervention development, a list of eligible and interested schools and colleges shall be prepared. The students, parents and teachers from these schools and colleges shall be requested to express their interest in participating in the FGDs and IDIs. The participants for the FGDs and IDIs shall be selected sequentially using a computer generated randomization process. The recruitment shall be stopped after reaching the data saturation for different stakeholder groups. The mental health and public health experts shall be identified through the existing research and academic networks of the project PIs. Additional participants shall be identified using snowballing techniques with help of the recruited participants. The recruitment shall be stopped after reaching the data saturation for different stakeholder groups.

For feasibility and validity testing, a list of eligible and interested schools and colleges shall be prepared. The youth, their parents and teachers from these schools and colleges shall be requested to express their interest in participating in the validity testing. A random selection method using a computer-generated selection shall be used to pick a proportionate random sample to recruit students based on grade levels and gender representation from among the list of the eligible and interested schools and colleges at each site. A random selection method using a computer-generated selection shall be used to pick a proportionate random sample of parents and teachers.

The different phases of the study are summarised in **Table 1**.

**Table 1:** Summary of the different phases of the study.

Phase	Participants	Methods	Output
Intervention development	Youth (children, adolescents, young adults), parents, teachers, experts	Literature review, FGDs, IDIs, NGT	Draft modular intervention package
Feasibility testing	Small sample (youth, parents, teachers)	Pilot delivery, qualitative and quantitative feedback	Refined intervention
Validation	Larger sample (youth, parents, teachers)	Pre-post assessments, process evaluation	Validation data

### 2.6 Intervention development and validation process

The intervention will consist of a comprehensive package of prevention- focused interventions targeted at youth (children, adolescents, young adults), parents and teachers for excessive and problematic use of digital technology and mitigate the risk of associated stress, depression, anxiety, addiction among youth. The package of interventions shall be developed using a co-creation approach with various stakeholders including the youth, teachers, parents, and mental health professionals, public health experts, and program or policy experts using the six steps in quality intervention development (6SQuID) framework (66) and new framework for developing and evaluating complex interventions (67). These frameworks will guide problem definition, identification of modifiable factors, selection of change mechanisms, delivery strategies, testing, refinement, and evaluation.

#### 2.6.1 Theoretical underpinnings

The intervention will be theoretically grounded in established psychological and behavioural frameworks, including the transtheoretical model of health behaviour change, social cognitive theory, theory of planned behaviour, self-determination theory, Knowles’ theory of adult learning, and the theory of reasoned action. These theories collectively inform the selection of cognitive, behavioural, and motivational strategies aimed at enhancing awareness, self-monitoring, decision-making, and sustained behaviour change. In addition, the strategies recommended in the previously published literature and as recommended by the stakeholders that are grounded in other theoretical constructs shall also be used.

The goals of the intervention are to promote responsible digital technology use, reduce risk factors for problematic use, enhance coping and self-regulation skills, foster healthy offline routines, and strengthen the role of parents and teachers as facilitators of digital well-being.

#### 2.6.2 Procedure

The development and testing of the intervention shall be based on the six steps in quality intervention development (6SQuID) framework and the new framework for developing and evaluating complex interventions (23–25). These shall include Step 1- defining and understanding the problem and its causes; Step 2- clarifying which contextual factors are malleable and have greatest scope for change; Step 3- identifying how to bring about change: the change mechanism; Step 4- identifying how to deliver the change mechanisms; Step 5- testing and refining on small scale; Step 6- collecting sufficient evidence of effectiveness to justify rigorous evaluation/implementation.

#### 2.6.3 Development of the intervention

The Steps 1–2 shall include a comprehensive analysis of the nature and extent of the problem and contributory factors. It shall be facilitated using desk review of existing literature and stakeholder participations. The existing research on the challenges encountered with the use of technology (e.g., impaired control over frequency, duration, and context of use) shall be searched. The review will also explore the contributory factors to these challenges. These would include psychological, behavioural, and environmental factors. This shall help identify key areas of focus for interventional components. Additionally, it will identify evidence-based interventional strategies that have been previously evaluated for their effectiveness in promoting responsible technology use and mitigating associated risks.

Stakeholder interviews will intend to understand the specific challenges and concerns related to digital technology use from the perspectives of key stakeholders. FGDs and IDIs will be conducted with each type of stakeholder to gather qualitative insights into their experiences, perceptions, and difficulties in managing technology use.

The qualitative findings from FGDs and IDIs will play a key role in the development of the intervention package. Thematic analysis will identify key challenges, behavioural patterns, contextual factors, and stakeholder priorities related to digital technology use. These themes will be mapped onto modifiable risk and protective factors. This will guide the identification of intervention targets and strategies.

Step 3 and 4 shall include identifying how to bring about change i.e. the change mechanism and identifying how to deliver the change mechanisms. An expert panel shall be constituted for finalization of the interventions for the package. This shall involve review of the information captured from the desk review and stakeholder interviews by a panel of the experts including psychiatrists, psychologists, technology experts, educationists, public health experts. The goal shall be to review the insights from the stakeholder interviews and existing literature to identify key strategies and interventions to be included in the package. A structured Nominal Group Technique (NGT) approach shall be used for this purpose.

A framework for intervention delivery shall be developed. The intervention package will comprise multiple, role-specific components for youth, parents, and teachers, developed through stakeholder consultations and expert consensus. This will involve systematically categorizing the interventions recommended by experts into a multi-tiered intervention delivery framework. The framework will be structured into four sets of intervention- youth set (children, adolescents, young adults), parental set, teacher set, collaborative set. The intervention package will be designed for practical implementation. This can be used on an as-needed basis. This will allow its integration into their daily routines effectively. The package shall be developed in a modular format. This will allow the use of different components on as- needed basis across diverse settings. The outcome will be a comprehensive, adaptable package of interventions.

#### 2.6.4 Feasibility assessment

Step 5 shall include testing and refining intervention on a small scale. The feasibility assessment of the intervention shall be carried out using a quasi-experimental, mixed-methods approach. Participants will be briefed in advance that this phase constitutes a feasibility and formative feedback assessment of a newly developed intervention package. Each group will be offered the interventions designed specifically for them (youth, parents, and teachers).Participants will evaluate each intervention on a Likert scale for clarity, perceived effectiveness, usefulness, and practicality. Additionally, a semi-structured questionnaire will gather qualitative feedback regarding their experiences, challenges, and suggested improvements. Students, parents and teachers will provide separate feedback on the individual interventions. The outcome of this step will be a set of feedback and recommendations regarding required changes to the package of prevention- focused interventions. The findings will be documented and shared with the expert panel for review. The experts will deliberate upon the findings and recommend the modifications for the intervention package.

#### 2.6.5 Intervention validation

Step 6 shall focus on collecting the validation data to justify rigorous evaluation/implementation.

The interventions shall be evaluated using a quasi-experimental, mixed-methods pre–post design. This shall be focused on validation, feasibility, and process outcomes. Base-line assessments shall be carried out using quantitative and qualitative measures. These shall include Structured proforma for socio- demographic details, Structured proforma for details of use of digital devices and internet, Smartphone Addiction Scale- Short Version (26), The Internet Addiction Test (IAT)- 6 item (27), Bergen Social Networking Addiction Scale (28), MULTICAGE CAD 4 (29), The Internet Gaming Disorder Scale-Short-Form (IGDS9-SF) (30), Gaming Disorder and Hazardous Gaming Scale (GDHGS) (31), Brief Biosocial Gambling Screen (BBGS) (32), Screen time Questionnaire. Participants from each group will receive the interventions designed specifically for them. young adults), parents, and teachers). The investigators on the project, project staff and other experts shall offer the intervention to the participants. Post- intervention assessments shall be carried out using quantitative and qualitative measures. These shall be guided by the Recommendations made in the new framework for developing and evaluating complex interventions and Moore’s framework for process evaluation.

Standard scoring procedures will be used for all instruments. Established cut-off scores will be used to classify the severity of problematic use (for example, SAS-SV and IAT-6). Other scales shall be interpreted using polythetic criteria and established scoring approaches consistent with prior validation studies (for example, BSNAS and IGDS9-SF). For scales where universally accepted cut-offs are not available, continuous scores will be analysed.

**Figure 1:**
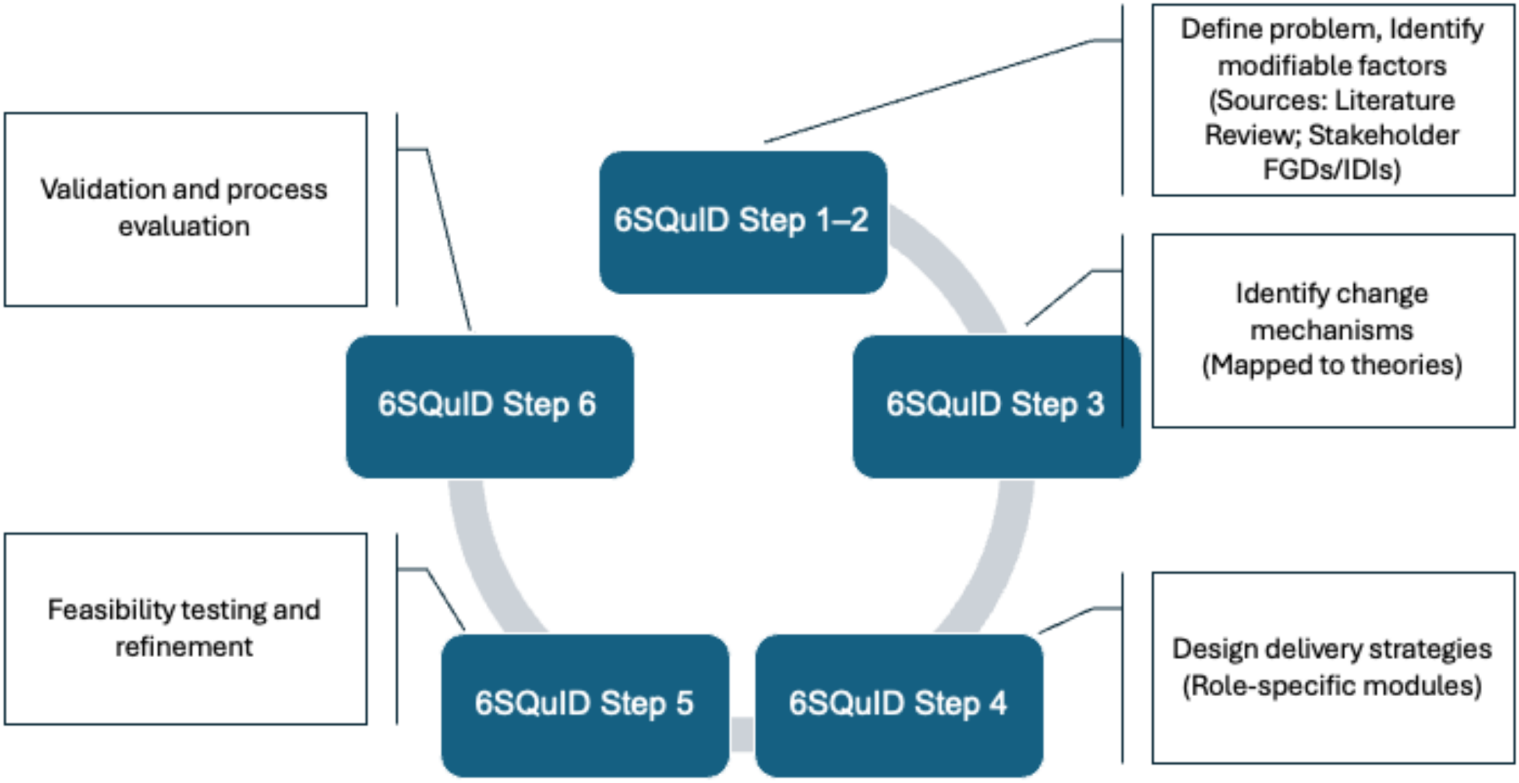
Various steps involved in the process based on the six steps in quality intervention development (6SQuID) framework.

Various steps involved in the process have been summarised in **Figure 1**.

Baseline assessments will be conducted immediately prior to the delivery of the intervention. Post-intervention assessments will be conducted immediately after completion of the intervention package. Long-term follow-up assessment is not planned in the present validation phase, as the focus is on feasibility, acceptability, and short-term proximal outcomes.

#### 2.6.7 Outcome measures

Recommendations made in the new framework for developing and evaluating complex interventions and Moore’s framework for process evaluation shall be used to interpret outcome results by examining what happened during implementation, to explain variation in outcomes across settings or populations and to support replication, adaptation, and scaling of interventions.

Moore’s framework for process evaluation of complex interventions provides a structured approach to understanding whether an intervention works. Also, it helps understand how and why it produces change. The framework emphasizes mechanisms of impact (e.g., changes in knowledge, skills, competence), implementation processes (e.g., fidelity, reach, quality), contextual influences, and short- and long-term outcomes. The present study aims to validate a newly developed, multi-component prevention package. The mapping of outcomes to Moore’s domains allows systematic examination of mechanisms driving behaviour change, implementation feasibility, and contextual variability across sites. This approach is particularly relevant for complex, multi-stakeholder interventions implemented in real-world educational settings.

The primary outcomes shall be the perceived change in knowledge, skill and confidence levels, and improvement in decision-making. The secondary outcomes shall include satisfaction with the intervention package; feasibility and acceptability of the intervention package; perceived long-term impact. In addition, contextual and implementation variables such as participation, context, fidelity and quality, engagement and motivation, and unintended effects shall also be assessed.

A mapping of these outcome measures to specific domains has been presented in **Table 2**. The primary and secondary outcomes of the study were mapped to specific domains of Moore’s framework. This was done with an aim to ensure conceptual alignment between intervention mechanisms, implementation processes, and measurable outcomes. Knowledge, skills, and confidence were categorized under mechanisms of Impact. Decision-making and perceived long-term impact were mapped under outcomes. Feasibility, acceptability, satisfaction, and contextual variables were mapped to implementation.

**Table 2:** Study outcomes/variables mapped to Moore’s domains.

Outcomes/Variables	Domain	Description
Primary outcomes		
Knowledge	Mechanisms of impact (Declarative Knowledge)	Understanding of healthy technology use, awareness of risks, knowledge of strategies
Skills	Mechanisms of impact (Procedural Knowledge)	Ability to apply strategies, feasibility of skills in real-life contexts
Confidence	Mechanisms of impact (Competence)	Self-efficacy and confidence in managing technology use
Decision-making	Outcomes	Changes in choices and self-regulation related to technology use
Secondary outcomes		
Satisfaction	Satisfaction	Overall satisfaction, perceived usefulness, willingness for future programs
Feasibility	Implementation plus context	Attendance feasibility, session length, applicability in real-life settings
Acceptability	Implementation plus satisfaction	Clarity, comfort, relevance, engagement
Perceived long-term impact	Outcomes	Sustainability, long-term benefit, likelihood of recommendation
Other variables		
Contextual and implementation variables	Participation (Reach/Dose)	Explains exposure and dosage effects
	Context	Explains variability across sites and populations
	Fidelity and quality	Distinguishes intervention vs implementation failure
	Engagement and motivation	Explains mechanisms driving behaviour change
	Unintended effects	Identifies unexpected positive/negative consequences

The assessments shall be carried out using quantitative and qualitative measures. These shall include structured questionnaires and FGDs and IDIs.

### 2.7 Statistical analysis plan

The data shall be analysed using quantitative and qualitative data analysis techniques. Descriptive statistics will be used to summarise socio-demographic characteristics, baseline patterns of digital technology use, and outcome measures for youth, parents, and teachers. Continuous variables will be reported as means with standard deviations or medians with interquartile ranges, as appropriate. Categorical variables will be presented as frequencies and percentages.

The clustering of participants within sites will be accounted for in the analysis. Mixed-effects models will be used. The study site will be included as a random effect to account for intra-site correlation and between-site variability.

For the validation phase, pre–post comparisons will be conducted within each participant group. Changes in primary and secondary outcome measures will be assessed using paired t-tests (Cohen’s d for paired samples) for normally distributed continuous variables and Wilcoxon signed-rank tests (effect size r) for non-normally distributed variables. For categorical outcomes, McNemar’s test (odds ratios or appropriate measures of association) will be applied. Where outcome measures are collected using multiple scales across domains of digital technology use, composite scores will be analysed according to standard scoring guidelines. Subgroup analyses may be conducted by age group, gender, and site to explore variability in outcomes. Missing data patterns will be examined prior to analysis. Sensitivity analyses may be conducted where appropriate. All statistical tests will be two-tailed, with a significance level set at p < 0.05.

Qualitative data from FGD, IDIs, and open-ended feedback questionnaires will be analysed using thematic analysis. Transcripts will be coded independently by at least two researchers to enhance credibility. Codes will be grouped into themes reflecting feasibility, acceptability, clarity, engagement, contextual influences, and perceived impact of the intervention. Discrepancies in coding will be resolved through discussion. An audit trail will be maintained.

A convergent mixed-methods approach will be used for data integration. Quantitative and qualitative data will be analysed in parallel during the feasibility and validation phases. Integration will occur at the interpretation stage. A triangulation approach will be used to compare findings across data sources. Convergence, complementarity, or divergence between quantitative and qualitative findings will be systematically examined.

Intention-to-treat (ITT) analysis will be used for the primary analyses. All participants enrolled in the study will be included in the analysis. This will be done irrespective of their level of participation or completion of the intervention. Missing data patterns will be examined (e.g., missing completely at random, missing at random, or not at random). Appropriate imputation techniques will be used to handle missing data. Sensitivity analyses (including complete-case analysis and comparison with imputed datasets) will be done to assess the robustness of findings.

The primary outcomes will be analysed without adjustment, as these are predefined and limited in number. For secondary outcomes and multiple sub-scale analyses, appropriate adjustments for multiple comparisons will be considered to control the risk of Type I error.

### 2.8 Implementation plan

The study will be initiated after obtaining the ethical clearance from the respective institutions involved. Afterwards, the schools and colleges interested in participation will be selected and they will be provided with consent and assent forms after briefing them about the research and its purpose. The school/college students, parent and teachers providing consent and assent (with parental consent) will then be selected for participation in the study. Mental health professionals, public health experts, and program or policy experts meeting the study inclusion and exclusion criteria shall be approached for participation and shall be included in the study after obtaining their consent. Data collection shall follow this. This shall include the development of intervention followed by feasibility assessment and validation. Data analysis shall be carried out subsequently.

Specific procedures will be used to ensure consistency in intervention delivery. Standardized intervention manuals, session plans, and training materials will be created and used at all sites. All facilitators involved in intervention delivery will undergo structured training and orientation prior to implementation. Fidelity checklists will be used during sessions to document adherence. Periodic monitoring will be conducted by the central research team through review of session records. Feedback will be provided to sites to address any deviations and ensure corrective action.

### 2.9 Data management and monitoring

All data will be handled with a uniform and secure process across sites. Each site will enter data into a coded database (using REDCap) with no personal identifiers. Records will be checked at the point of entry for missing fields, range errors, and inconsistencies. A central team will review weekly uploads from all sites to ensure uniform coding, consistent variable labels, and correct scoring of all study tools. Any errors identified during this review will be sent back to the site for correction.

Data will be stored on secure, access-controlled servers. Only approved members of the study team will have access to the full dataset. Each site will keep a separate record of assent and consent forms, and these will not be linked to the main dataset. Monitoring will include regular checks for outliers, and unusual response patterns. These checks will ensure that the dataset remains stable. A central monitor will track recruitment numbers, data quality, and protocol adherence. Sites will report any deviation from procedures at once. All steps in data handling will follow good research-practice standards and adhere to the requirements of the ethics approvals granted for the study.

### 2.10 Ethics procedure

The study shall obtain approval from the institutional ethics committee prior to commencement. Informed assent/consent will be obtained from the participants and, informed consent from parents or legal guardians will be obtained for minors. Participants will have the right to withdraw their assent/consent during the study without providing any justification. No personally identifiable information will be gathered. The collected data will be safely kept and preserved. Anonymization techniques will be used to prevent any potential re-identification of the participants. The research report will not disclose the identity of the participants.

## 3 Results

At the time of manuscript submission, data collection has not yet commenced. The findings of the study shall be reported following the data analysis.

## 4 Discussion

This study protocol describes the development and validation of a prevention-focused intervention package targeted at problematic use of digital technology among youth. The intervention also aims to address the associated stress, depression, anxiety and addiction among the youth. The approach is relevant in context of the growing exposure of children, adolescents, and young adults to digital devices and the lack of standardized, preventive frameworks tailored to this population in India. Problematic use of digital technology among the youth has been reported from the country in various studies (33–35). The focus on early prevention is in keeping with evidence showing that a large proportion of mental and behavioural disorders begin during adolescence and young adulthood (36). This highlights the importance of interventions before clinical thresholds are reached. Prevention focused interventions have been found to be effective in improving psychosocial outcomes among young people (37, 38). This is particularly relevant to the LMIC.

This study will use a structured and theory-based method to develop and validate the interventions. The established frameworks for complex intervention development will provide a clear stepwise approach to the process. This will include defining the problem, identifying change targets, selecting strategies, and refining delivery. This will strengthen design and support reproducibility.

The co-creation approach will be a key strength of the study. Involvement of youth, parents, teachers, mental health experts, addiction experts, child and adolescent psychiatry experts, public health experts, and policy and program experts will help ensure that the intervention package reflects real-world needs. This is expected to increase relevance, feasibility, and acceptance across schools, colleges, and families (39, 40). Educational settings offer a scalable platform for preventive interventions (41–43). Also, engagement of parents is particularly relevant for digital technology use as it spans home and educational environments. Thus, it is expected to be impacted by the shared norms and supervision (44–46).

Refinement of the intervention package before scale-up will be supported by the phased design of the study. Qualitative methods will be used to guide the development. The outcomes and process measures will be documented using the mixed-methods approach (47, 48). This will ensure clear assessment of feasibility, acceptability, and engagement (49, 50).

Multi-site implementation will strengthen external validity. The modular structure will allow flexible use of components. The prevention focus will address the risk early and support public health action before harm occurs.

### 4.1 Expected outcomes

A validated intervention package designed for youth, parents, and teachers shall be developed at the end of the study. The package will be based on the established theories and evidence. Also, it shall be guided by the stakeholder input. The primary expected outcomes of the study include improved knowledge of healthy digital technology use, stronger self-regulation skills, increased confidence in managing digital behaviour, and more mindful decision-making related to use of digital technology.

The feasibility, acceptability, and perceived usefulness of the intervention package will be established during the validation phase. Assessment of changes in awareness, skills, and decision-making will be done using the pre-post design. Process related data will provide information about participant engagement, fidelity of delivery, and contextual influences. The findings will inform future evaluations at a larger scale. This phase is not intended to establish causal effectiveness.

### 4.2 Public health and policy relevance

The intervention package will help intervene in the context of digital technology use before it reaches clinical severity. Currently, there are limited prevention focused interventions targeted at addictive behaviours in India. This will support population-level digital and overall mental wellbeing. The inclusion of parents and teachers shall strengthen shared responsibility. This shall also support consistent messaging across home and educational environments.

The modular design of the intervention will allow flexible use across schools, colleges, home and community settings. The package can be integrated into existing school health initiatives, life skills education, and teacher training programs. Findings from this study may guide the development of practical guidance for safe and healthy technology use among youth at regional and national levels. The intervention shall be in line with the national mental health policy and National Education Policy (NEP 2020) of India (51, 52).

### 4.3 Anticipated challenges

Differences in access to digital devices may affect implementation across sites. Rapid changes in digital platforms may reduce long-term relevance of specific examples, requiring periodic updates to content. Self-reported measures may introduce bias, though mixed methods will provide contextual depth.

The quasi-experimental design limits causal inference. The study focuses on validation and readiness for scale-up rather than effectiveness testing. These limits are consistent with the aims of a development and validation protocol. A more extensive evaluation is planned following the implementation of the interventions in the real world settings at a larger scale.

### 4.4 Study status and timeline

Ethical approvals have been obtained, and site preparation is complete. Data collection has not yet started. The study will proceed in phases. It shall begin with intervention development. This will be followed by feasibility testing and validation.

## 5 Conclusion

This protocol outlines the process of development of a prevention focused intervention package targeted at problematic digital technology use and associated stress, depression, anxiety and addiction among the youth. The study emphasizes early intervention that is guided by active stakeholder engagement. The resulting intervention is expected to support scalable prevention efforts. It is also expected to guide the future policy actions focused on youth digital well-being. This work shall contribute towards ensuring “Digital Wellness for All” by promoting safe and healthy use of digital technology.
